# A New Effect Size for Meta‐Analysis of Magnitude: lnM

**DOI:** 10.1111/ele.70428

**Published:** 2026-09-03

**Authors:** Shinichi Nakagawa, Ayumi Mizuno, Coralie Williams, Malgorzata Lagisz, Rose E. O'Dea, Daniel W. A. Noble, Alistair M. Senior, Erick Lundgren, Santiago Ortega

**Affiliations:** ^1^ Collaboration for Open Science and Synthesis in Ecology and Evolution (COSSEE), Department of Biological Sciences, Faculty of Science University of Alberta Edmonton Canada; ^2^ Evolution and Ecology Research Centre, School of Biological, Earth and Environmental Sciences University of New South Wales Sydney New South Wales Australia; ^3^ School of Mathematics and Statistics The University of New South Wales Sydney New South Wales Australia; ^4^ Division of Ecology and Evolution Research School of Biology, The Australian National University Canberra Australia; ^5^ School of Life and Environmental Sciences The University of Sydney Sydney New South Wales Australia; ^6^ Charles Perkins Centre The University of Sydney Sydney New South Wales Australia; ^7^ Sydney Precision Data Science Centre The University of Sydney Sydney New South Wales Australia

**Keywords:** Bayesian statistics, biological optima, coefficient‐of‐variation ratio, location‐scale models, meta‐regression

## Abstract

Meta‐analyses in ecology and evolution sometimes target the magnitude of differences between groups rather than their direction, for example, when the question concerns deviation from a biological optimum or divergence from a reference state. A common practice is to convert signed effects into magnitudes by taking absolute values, but this can induce upward distortion and non‐normal sampling distributions under standard meta‐analytic models. Here we introduce lnM, a log‐ratio effect size for the magnitude of difference between two groups, defined from one‐way ANOVA components. lnM is asymptotically normal, supports standard multilevel meta‐analysis and meta‐regression and applies to both ratio‐ and interval‐scale traits. Using theory, simulations and worked examples, we show when delta‐method and single‐fit bootstrap estimators perform well, and how lnM can be used to assess publication bias.

## Graphical abstract

Meta‐analyses often ask how strongly groups differ, irrespective of direction, but converting signed effects to absolute values can inflate magnitudes and produce folded, non‐normal sampling distributions. We introduce lnM, a log‐ratio of between‐group to within‐group standard deviations, to quantify direction‐free separation on an approximately normal scale. By re‐expressing small separations as negative values and large separations as positive values, lnM better separates magnitude patterns across datasets, supports multilevel meta‐analysis and meta‐regression, and reveals small‐study effects. Simulations and ecological reanalyses show that lnM, especially with single‐fit bootstrap estimation, provides a practical framework for synthesising magnitude.

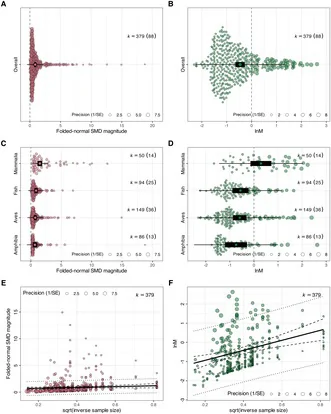

## 1 Introduction

Meta‐analysis serves a unique purpose by allowing researchers to synthesise parameters and uncover broad patterns across diverse studies that single‐data analyses cannot achieve (Arnqvist and Wooster 1995; Gurevitch et al. 2018). Standardised (unit‐free) effect sizes that compare two groups, such as standardised mean differences (SMD; its estimators, known as Cohen's d or Hedges' g) and log response ratios (lnRR), now underpin thousands of quantitative reviews (Hedges 1981; Hedges et al. 1999). Newer variance‐based metrics, such as the log variability ratio (lnVR) and the log coefficient of variation ratio (lnCVR), have extended this toolkit to address differences in within‐group variation (Nakagawa et al. 2015). Despite these advancements, we still lack a widely adopted effect size specifically designed to isolate the magnitude of a difference; that is, how far apart two groups are, irrespective of direction (cf. Morrissey 2016b; Kulinskaya and Hoaglin 2023). Magnitude is often more biologically meaningful than the sign of an effect, particularly when researchers investigate deviations from biological optima or population divergence, where the absolute size of the change from a reference state is the critical parameter to measure in ecological contexts, rather than the direction (e.g., Kingsolver et al. 2001) (reviewed in Morrissey 2016b).

In practice, investigators often circumvent this gap by using the absolute value of signed effects (i.e., ∣lnRR∣ or ∣SMD∣). By mapping both positive and negative sampling deviations onto the same positive scale, absolute values tend to inflate overall effect magnitudes; this is especially true when effects are small, relative to sampling error and particularly worse when sampling error is not accounted for (Nakagawa and Lagisz 2016). In addition, these transformed effect sizes are bounded at zero and have folded, non‐Gaussian sampling distributions, making them awkward to analyse within standard meta‐analytic frameworks. Consequently, meta‐analyses based on absolute transformations can complicate inference about, for example, the strength of selection, the magnitude of ecological impacts, or treatment efficacy (Morrissey 2016a, 2016b). To reduce this problem, Morrissey (2016b) proposed an analysis strategy, whereby models are first fitted to signed effects and only the resulting estimates are converted to magnitudes. Although this can recover unbiased overall means, it is not readily extended to flexible meta‐regression with multiple moderators (both continuous and categorical) and has therefore seen limited practical uptake.

Recently, Kulinskaya and Hoaglin (2023) developed a principled framework for unsigned mean‐based effects. They clarified the distributional basis of absolute mean differences and absolute SMDs (∣d∣), relating ∣d∣ to folded‐t and $d^{2}$ to noncentral F distributions, and provided methods for estimation and interval construction under random‐effects meta‐analytic models. This work represents an important advance over naive absolute transformations. However, for applied ecologists, a practical limitation remains; these unsigned metrics are still not easily accommodated within standard linear meta‐regression frameworks, particularly when one wishes to model multiple moderators, including continuous predictors.

To resolve the above‐mentioned problems, we propose an effect size that is often more biologically meaningful, the “magnitude of separation” between groups relative to within‐group noise, irrespective of direction. We introduce the log‐magnitude effect size, lnM, which targets the magnitude of mean separation relative to within‐group variation on a log scale. Importantly, lnM is compatible with standard meta‐analytic models (cf. Cinar et al. 2022; Williams et al. 2025) and it supports meta‐regression with multiple categorical or continuous moderators. By operating on the log scale, lnM avoids the zero‐boundary problem, enabling inference on a (nearly) Gaussian, unbounded scale, that remains interpretable for both ratio and interval data. For cases in which lnM cannot be defined analytically (a key limitation described below), we show that a single‐fit parametric bootstrap provides reliable point estimates and sampling variances (Nakagawa et al. 2026). Further, we describe how to interpret lnM, whose 0 (when between‐group separation and within‐group variation are the same) is approximately equivalent to d=1.4; this equivalence may feel counterintuitive and surprising, as a d of 1.4 is very large (further discussed in Section 2.5).

The remainder of the paper proceeds as follows. First, we connect historical motivations for comparing magnitudes to Lynch's variance‐ratio framework and recent meta‐analysis of variation (Lynch 1990; Nakagawa et al. 2015; Senior et al. 2020). We then derive lnM and its sampling variance, validate its performance via simulation, and describe its interpretation and connection to d (SMD). Finally, we re‐analyse two published datasets of ecological examples to illustrate how lnM delivers regression‐friendly magnitude estimates, contrasting to absolute‐value approaches. An online tutorial illustrates R (https://itchyshin.github.io/meta‐analysis_of_magnitude/) implementation using metafor (Viechtbauer 2010), and helper functions in orchaRd (Nakagawa et al. 2021, 2023).

## 2 Developing a New Effect Size: lnM

### 2.1 Inspiration

Interest in the distance between two ecological or evolutionary groups, regardless of the sign of the difference, has a long history in evolutionary biology (Haldane 1949). Notably, Lynch (1990) proposed the ratio of between‐to within–population variance as a scale‐free yardstick for morphological divergence under a neutral model of evolution. More recently, the log‐variability ratio (lnVR) and its derivative, along with the log‐coefficient‐of‐variation ratio (lnCVR), were proposed to meta‐analyse differences in variability (sample standard deviations) between two groups (Nakagawa et al. 2015; Senior et al. 2020). Drawing on these foundations, we develop the log‐magnitude statistic, lnM. This effect size maintains an intuitive link to Lynch's variance‐ratio, which is clearly inspired by classical ANOVA, but with key methodological refinements. First, by working on the log scale, lnM converts the ratio of variance components into an asymptotically normally distributed quantity. Second, unlike Lynch's ratio (which compares variances), lnM compares standard deviations (the square root of variance), which is more comparable to lnVR, the ratio of two standard deviations. Also, whereas Lynch's metric requires natural‐log‐transformed (ln) sample means and variances, lnM is calculated using statistics on the original scale (see below).

### 2.2 Definition and Extension

We define lnM as an effect size for the *magnitude* of a two‐group difference (how far apart two groups are), irrespective of direction. Let the observed summary statistics be $\left({\bar{X}}_{1},s_{1},n_{1}\right)$ for group 1 and $\left({\bar{X}}_{2},s_{2},n_{2}\right)$ for group 2; these are the same basic inputs required for SMD and lnRR. For a two‐group comparison, the classical one‐way ANOVA identities provide convenient building blocks. The between‐ and within‐group mean squares are

(1) $$MS_{B}=\frac{n_{1}n_{2}}{n_{1}+n_{2}}\;{\left({\bar{X}}_{1}-{\bar{X}}_{2}\right)}^{2}$$

(2) $$MS_{W}=\frac{\left(n_{1}-1\right)s_{1}^{2}+\left(n_{2}-1\right)s_{2}^{2}}{n_{1}+n_{2}-2}$$

We define the within‐group variance as $s_{W}^{2}=MS_{W}$. To obtain a between‐component on the original measurement scale, we subtract the within‐from the between‐group mean square, and rescale by the harmonic‐mean sample size $n_{0}=2n_{1}n_{2}/\left(n_{1}+n_{2}\right)$, defining (Lynch 1990):

(3) $$s_{B}^{2}\equiv \frac{MS_{B}-MS_{W}}{n_{0}}=\frac{MS_{B}-s_{W}^{2}}{n_{0}}$$

where $s_{B}^{2}$ is a function of the squared mean difference (i.e., the dispersion/separation between two point means around the grand mean).

We then define lnM as the log ratio of the between‐component (mean‐separation) standard deviation and within‐group standard deviation:

(4) $$\mathrm{lnM}\equiv \ln\left(\frac{s_{B}}{s_{W}}\right)$$

We now apply the first‐order Taylor expansion, also known as the delta method (Ver Hoef 2012), to derive the sampling variance for both independent and dependent cases (full derivations in the Appendix A). The sampling variance for two independent groups is as follows:

(5) $${\mathrm{Var}}_{\mathrm{IND}}\left(\mathrm{lnM}\right)=\frac{1}{4\Delta^{2}}\left[{\left(\frac{n_{0}}{2}\right)}^{2}\left(2\;s_{D}^{4}+4\;s_{D}^{2}\;\delta^{2}\right)+\frac{MS_{B}^{2}}{MS_{W}^{2}}\;\frac{2\;MS_{W}^{2}}{\;n_{1}+n_{2}-2\;}\right]$$

where $\Delta =MS_{B}-MS_{W}$, $n_{0}=2n_{1}n_{2}/\left(n_{1}+n_{2}\right)$ is the harmonic‐mean sample size, $\delta ={\bar{X}}_{1}-{\bar{X}}_{2}$ is the observed difference in group means and $s_{D}^{2}=s_{1}^{2}/n_{1}+s_{2}^{2}/n_{2}$ is the sampling variance of δ.

Similarly, the sampling variance for paired (dependent) groups is as follows:

(6) $${\mathrm{Var}}_{\mathrm{DEP}}\left(\mathrm{lnM}\right)=\frac{1}{4\Delta^{2}}\left[{\left(\frac{n}{2}\right)}^{2}\left(\frac{2\;s_{D}^{4}}{n^{2}}+\frac{4\;\delta^{2}\;s_{D}^{2}}{n}\right)+\frac{MS_{B}^{2}}{MS_{W}^{2}}\;\frac{s_{1}^{4}+s_{2}^{4}+2\;r^{2}s_{1}^{2}s_{2}^{2}}{2\;\left(n-1\right)}\right]$$

where n is the number of paired observations (i.e., $n_{1}=n_{2}=n$), r is the within‐pair correlation, $s_{D}^{2}=s_{1}^{2}+s_{2}^{2}-2\;r\;s_{1}s_{2}$ is the variance of the within‐pair difference, and $\delta ={\bar{X}}_{1}-{\bar{X}}_{2}$ is the corresponding mean difference (this correlation can be obtained from primary studies or often assumed to be r=0.5 or 0.8) (Noble et al. 2017; Pustejovsky and Tipton 2022). The quantity Δ again denotes $MS_{B}-MS_{W}$; for the paired design $MS_{B}=\left(n/2\right){\left({\bar{X}}_{1}-{\bar{X}}_{2}\right)}^{2}$ and $MS_{W}=\left(s_{1}^{2}+s_{2}^{2}\right)/2$.

These formulas highlight two practical features of lnM. First, the precision of lnM depends strongly on how far the between‐group mean square $MS_{B}$ sits above the within‐group mean square $MS_{W}$. As the delta‐method expressions show, $\mathrm{Var}\left(\mathrm{lnM}\right)$ scales approximately as ${\left(MS_{B}-MS_{W}\right)}^{-2}$. When $MS_{B}$ exceeds $MS_{W}$, standard errors shrink rapidly; as $MS_{B}$ approaches $MS_{W}$, the variance becomes large. Second, lnM becomes undefined when $MS_{B}\leq MS_{W}$. When $MS_{B}=MS_{W}$, the estimated between‐group variance is zero and lnM approaches −∞. In practice, such cases are common: whenever group means are similar, a non‐trivial fraction of contrasts will fall into this undefined or numerically unstable region. Discarding these cases would systematically remove studies with small separations, a limitation that likely restricted the adoption of Lynch's rate‐of‐evolution index (Lynch 1990), which relies on a similar variance‐ratio construction. In the next section, we address both issues by introducing a simulation‐based approach, below, that stabilises lnM, providing finite estimates and standard errors even when $MS_{B}\leq MS_{W}$.

While the closed‐form variance formulas are convenient, they have two limitations. First, when sample sizes are small, the first–order Taylor approximation can be imprecise (e.g., Senior et al. 2020). Second, as noted earlier, when the difference $\Delta =MS_{B}-MS_{W}$ is non‐positive, lnM becomes undefined. A practical remedy is the single–fit parametric bootstrap, originally introduced by Mandel (2013) and Fletcher and Jowett (2022). This single‐fit bootstrap is faster and simpler than traditional bootstrap methods (Tibshirani and Efron 1993; Davison and Hinkley 1997). More recently, Nakagawa et al. (2026) integrated this method into SAFE; Single‐fit, Accurate, Fast and Easy bootstrap to obtain bias‐corrected point estimates and sampling variances for effect‐size statistics. SAFE replaces complex algebra with four vectorised steps that run in milliseconds: (1) fit once, (2) draw once, (3) transform once and (4) summarise twice.

The method leverages standard distributional theory: we assume sample means $\left({\bar{X}}_{1},{\bar{X}}_{2}\right)$ are normally distributed, while sample variances follow a $\chi^{2}$ (chi‐squared) distribution $\left(s_{1}^{2},s_{2}^{2}\right)$ (Anderson 2003). The $\chi^{2}$ distribution arises whenever we take the sum of squared normal variables; in fact, $\left(n-1\right)s^{2}/\sigma^{2}∼\chi_{n-1}^{2}$ whenever data are independently and normally distributed with true variance $\sigma^{2}$. For multivariate settings, we employ the Wishart distribution, the multivariate analogue of the $\chi^{2}$ distribution. If we collect p correlated normal variables, their sample covariance matrix follows a Wishart distribution, denoted $W_{p}\left(\Sigma ,\mathrm{df}\right)$, where the true covariance matrix Σ plays the role of $\sigma^{2}$, and the degrees of freedom df depend on the sample size. These distributional facts guarantee that simulated variance draws are always positive and respect the independence between means and variances under normal sampling. Using SAFE we can then estimate lnM and its sampling variance under these distributional assumptions as follows.

**Step 1: Fit once**. We begin by taking the observed pair of sample statistics for which we wish to calculate lnM $\left({\bar{X}}_{1},{\bar{X}}_{2},s_{1}^{2},s_{2}^{2}\right)$ and sample sizes $\left(n_{1},n_{2}\right)$ (and correlation r for paired data). For independent groups, we assume the sample mean and variance for the two groups are distributed as:

(7) $${\bar{X}}_{g}^{*}∼N\left({\bar{X}}_{g},\frac{s_{g}^{2}}{n_{g}}\right),\;\;\frac{\left(n_{g}-1\right)\;S_{g}^{2*}}{s_{g}^{2}}∼\chi_{n_{g}-1}^{2},\;g=1,2$$

where ${\bar{X}}_{g}^{*}$ is independent of $S_{g}^{2*}$. Note that drawing from these distributions (Step 2, below) produces four vectors of replicates, which we refer as ‘clouds’ (bootstrapped vectors): ${\left({\bar{X}}_{1}^{*},{\bar{X}}_{2}^{*},s_{1}^{2*},s_{2}^{2*}\right)}^{⊤}$ (i.e., quadruplets of clouds).

For paired groups ($n_{1}=n_{2}=n$), we define the covariance matrix:

(8) $$\Sigma =\left[\begin{array}{cc} s_{1}^{2} & rs_{1}s_{2}\\ rs_{1}s_{2} & s_{2}^{2} \end{array}\right]$$

The joint distributions for both means and variances are then:

(9) $${\left({\bar{X}}_{1}^{*},{\bar{X}}_{2}^{*}\right)}^{⊤}∼N\left({\left({\bar{X}}_{1},{\bar{X}}_{2}\right)}^{⊤},\frac{\Sigma }{n}\right),\;\;\left(n-1\right)\;S^{*}∼W_{2}\left(\Sigma ,n-1\right)$$

independently, where $S^{*}$ is a 2×2 covariance matrix. In practice, we only use its diagonal entries $S_{11}^{*}$ and $S_{22}^{*}$ to form $MS_{W}$.

**Step 2: Draw once**. We next simulate a large Monte Carlo batch, say B=10,000 clouds, from the normal–$\chi^{2}$/Wishart distributions, as above. This produces surrogate means and variances that mimic repeated sampling from the underlying populations. In practice, this step can be done for independent cases with the R functions rnorm and rchisq, while dependent cases require mvrnorm from the MASS package (Venables and Ripley 2013), and the base function rWishart.

**Step 3: Transform once**. We convert every surrogate draw into a value of ${\mathrm{lnM}}^{*}$ by the same algebra that defines Equations ((1), (2), (3), (4)–(1), (2), (3), (4)). Crucially, we retain only those replicates where $MS_{B}^{*}>MS_{W}^{*}$.

**Step 4: Summarise twice**. ‘Twice’ means we obtain two quantities: a bias‐corrected (BC) point estimate and standard error (the square root of sampling variance). Having obtained a bootstrap sample ${\left\{{\mathrm{lnM}}^{*\left(i\right)}\right\}}_{i=1}^{B}$, these can be then estimated by:

(10) $${\mathrm{SE}}_{\text{SAFE}}\left(\mathrm{lnM}\right)=\sqrt{{\mathrm{Var}}_{\text{SAFE}}\left(\mathrm{lnM}\right)}=\sqrt{\frac{1}{B-1}\sum_{i=1}^{B}{\left({\mathrm{lnM}}^{*\left(i\right)}-\bar{{\mathrm{lnM}}^{*}}\right)}^{2}}$$

(11) $$\text{bias}=\bar{{\mathrm{lnM}}^{*}}-{\mathrm{lnM}}_{\mathrm{PI}}$$

(12) $${\mathrm{lnM}}_{\mathrm{BC}}={\mathrm{lnM}}_{\mathrm{PI}}-\text{bias}=2{\mathrm{lnM}}_{\mathrm{PI}}-\bar{{\mathrm{lnM}}^{*}}\left(={\mathrm{lnM}}_{\text{SAFE}}\right)$$

where $\bar{{\mathrm{lnM}}^{*}}$ is the average of bootstrap values (i.e., $\bar{{\mathrm{lnM}}^{*}}=\frac{1}{B}\sum_{b=1}^{B}{\mathrm{lnM}}^{*\left(b\right)}$) and ${\mathrm{lnM}}_{\mathrm{PI}}$ is a plug‐in estimate obtained from Equation (4).

Bias in the plug‐in lnM estimate arises because lnM is a nonlinear function of the sample means and variances, so $E\left[{\mathrm{lnM}}_{\mathrm{PI}}\right]\neq {\mathrm{lnM}}_{\text{true}}$ in finite samples; in other words, the plug‐in value of lnM is biased in relation to the true value of lnM. The bootstrap cloud $\left\{{\mathrm{lnM}}^{*\left(i\right)}\right\}$ directly approximates the true sampling distribution of lnM, and its mean minus the plug‐in value provides an empirical estimate of that systematic error. Subtracting this estimated bias yields the bias‐corrected point estimate ${\mathrm{lnM}}_{\mathrm{BC}}$, which restores accuracy even when samples are small or in cases where the delta approximation is poor (see the online supplement for the full implementation of SAFE in R). Furthermore, because SAFE samples directly from the finite‐sample distribution of the four summary statistics $\left({\bar{X}}_{1},{\bar{X}}_{2},s_{1}^{2},s_{2}^{2}\right)$, its standard error and bias correction remain valid when n is small, when the delta approximation is poor, or when Δ is close to zero.

However, the issue we mentioned earlier is that ${\mathrm{lnM}}_{\mathrm{PI}}$ is not always definable (Equation (4)). In such a case, we define lnM as the mean of the valid bootstrap replicates:

(13) $${\mathrm{lnM}}_{\text{SAFE}}=\bar{{\mathrm{lnM}}^{*}}\;\text{if}\;MS_{B}\leq MS_{W}$$

Therefore, even in the problematic scenario where $MS_{B}\leq MS_{W}$ (for which the analytic lnM is undefined), SAFE still returns a usable estimate and standard error (SE), provided that at least some bootstrap replicates satisfy $MS_{B}^{*}>MS_{W}^{*}$. Although the accuracy of ${\mathrm{lnM}}_{\text{SAFE}}$ as in Equation (13) cannot be validated, we expect these values to be smaller than all lnM values that can be estimated without the bootstrap. We now use simulation to compare the performance of the SAFE approach with that of the closed‐form formulas, including the behaviour of ${\mathrm{lnM}}_{\text{SAFE}}$ values (i.e., Equation 13).

### 2.3 Simulation Design

We simulated samples for two groups and estimated the sample mean and standard deviation for group 1 and group 2 and the correlation between the two groups: $\left({\bar{X}}_{1},s_{1},{\bar{X}}_{2},s_{2},r\right)$. In our simulation, we denote true (population) values of these statistics as $\left(\mu_{1},\sigma_{1},\mu_{2},\sigma_{2},\rho \right)$ respectively. We closely followed the style of a simulation study used by Senior et al. (2020), who studied the performance of the delta‐method‐based formulas for lnRR, lnVR and lnCVR (cf. Lajeunesse 2015); we also followed Williams et al. (2024) for designing and reporting, where possible.

For every run, we drew two vectors of length $n_{1}$ and $n_{2}$ (sample size for group 1 and group 2) from a bivariate normal distribution

(14) $$\left[\begin{array}{c} X_{1i}\\ X_{2i} \end{array}\right]∼N\left(\left[\begin{array}{c} \mu_{1}\\ \mu_{2} \end{array}\right],\left[\begin{array}{cc} \sigma_{1}^{2} & \rho \;\sigma_{1}\sigma_{2}\\ \rho \;\sigma_{1}\sigma_{2} & \sigma_{2}^{2} \end{array}\right]\right),\;\;i=1,\ldots ,\max\left(n_{1},n_{2}\right)$$

where ρ=0 gives independent groups and ρ=0.8 produces paired data for dependent groups. The population (‘true’) means were set to $\mu_{1}=0$ and $\mu_{2}=\theta$, with θ∈{0.2,0.3,0.4,0.5,0.6,0.7,0.8,0.9, 1,1.2,1.4,1.6,1.8,2,2.5,3,4,5} to span from negligible to large magnitudes. Throughout, the within‐group standard deviations were equal ($\sigma_{1}=\sigma_{2}=1$), ensuring that changes in lnM stem from the distance between means rather than from differences in dispersion (within‐group standard deviation); examining Equation (2) tells us differences in standard deviation between two groups do not have a major impact on lnM as the two sample standard deviations are weighted by their sample sizes. Because of this parameterization, our mean difference θ is mathematically equivalent to the true standardised mean difference (d), making it effectively a unit‐free scale in this specific context. Note that $\theta^{2}$ is equivalent to $s_{B}^{2}$ and $\left(\sigma_{1}+\sigma_{2}\right)/2$ is $s_{W}^{2}$ where lnM =$\;\ln\left(s_{B}/s_{W}\right)$.

For independent groups, balanced and unbalanced designs were examined with $\left(n_{1},n_{2}\right)$ chosen from {(5,5), (10,10), (20,20), (100,100), (3,7), (6,14), (12,28), (40,160)}. This was explored as unbalanced designs may impact lnM via changes in $s_{B}$ (cf., Equation 1). For dependent groups, we only selected balanced designs. Therefore, we have 144 parameter sets (combinations) for independent groups, while 72 sets for dependent groups (in total, 216 combinations).

For every replicate ($K=10^{5}$ per parameter set; 216 sets), we computed: (1) the plug‐in point estimates, either ${\hat{\mathrm{lnM}}}_{\mathrm{PI}}$ and its first–order delta–method variance ${\hat{\mathrm{Var}}}_{\mathrm{IND}}\left(\mathrm{lnM}\right)$ Equation (5) (independent groups) or ${\hat{\mathrm{Var}}}_{\mathrm{DEP}}\left(\mathrm{lnM}\right)$ Equation (6), collectively these are called ${\hat{\mathrm{Var}}}_{\text{delta}}\left(\mathrm{lnM}\right)$ (dependent groups; note that we added ‘hat’ symbols to these to make it clear that these are estimators) and (2) the safe bootstrap cloud ${\left\{{\mathrm{lnM}}^{*\left(b\right)}\right\}}_{b=1}^{B}$ with $B=10^{5}$ draws (note that we collected $10^{5}$ bootstrap draws regardless of the rate of discarded draws), together with its bias‐corrected mean ${\hat{\mathrm{lnM}}}_{\mathrm{BC}}$ and the bootstrap variance ${\hat{\mathrm{Var}}}_{\text{SAFE}}\left(\mathrm{lnM}\right)$. For each estimator ${\hat{\mathrm{lnM}}}_{l}\in \left\{{\hat{\mathrm{lnM}}}_{\mathrm{PI}},{\hat{\mathrm{lnM}}}_{\mathrm{BC}}\right\}$ we calculated the empirical bias:

(15) $$\text{bias}\left({\hat{\mathrm{lnM}}}_{l}\right)={\bar{\hat{\mathrm{lnM}}}}_{l}-{\mathrm{lnM}}_{\text{true}}$$

(16) $$\bar{{\hat{\mathrm{lnM}}}_{l}}=\frac{1}{K}\sum_{k=1}^{K}{\hat{\mathrm{lnM}}}_{l}^{\left(k\right)}$$

where ${\mathrm{lnM}}_{\text{true}}$ is defined by the population parameter values in Equations ((1), (2), (3), (4)–(1), (2), (3), (4)) and k denotes the k‐th simulation (k=1,2,…K; $K=10^{5}$). We can interpret this bias as the deviation of the estimator (${\mathrm{lnM}}_{l}$) from the true population value. For each variance estimator ${\hat{\mathrm{Var}}}_{l}\in \left\{{\hat{\mathrm{Var}}}_{\text{delta}},{\hat{\mathrm{Var}}}_{\text{SAFE}}\right\}$ we calculated the relative bias as follows:

(17) $$\text{Relative bias}\left({\hat{\mathrm{Var}}}_{l}\right)=\frac{\bar{{\hat{\mathrm{Var}}}_{l}}-{\mathrm{Var}}_{\mathrm{MC}}\hat{\left(\mathrm{lnM}\right)}}{{\mathrm{Var}}_{\mathrm{MC}}\hat{\left(\mathrm{lnM}\right)}}\times 100$$

(18) $$\bar{{\hat{\mathrm{Var}}}_{l}}=\frac{1}{K}\sum_{k=1}^{K}{\hat{\mathrm{Var}}}_{l}^{\left(k\right)}$$

(19) $${\mathrm{Var}}_{\mathrm{MC}}\hat{\left({\mathrm{lnM}}_{l}\right)}=\frac{1}{K-1}\sum_{k=1}^{K}{\left({\hat{{\mathrm{lnM}}_{l}}}^{\left(k\right)}-\bar{\hat{{\mathrm{lnM}}_{l}}}\right)}^{2}$$

${\mathrm{Var}}_{\mathrm{MC}}\hat{\left({\mathrm{lnM}}_{l}\right)}$ is a variance estimate based on all Monte Carlo simulation runs ($K=10^{5}$) for a combination of parameters, and ${\hat{\mathrm{lnM}}}_{l}\in \left\{{\hat{\mathrm{lnM}}}_{\mathrm{PI}},{\hat{\mathrm{lnM}}}_{\mathrm{BC}}\right\}$. Yet, we only used ${\hat{\mathrm{lnM}}}_{l}={\hat{\mathrm{lnM}}}_{\mathrm{BC}}$ as mentioned; ${\hat{\mathrm{lnM}}}_{\mathrm{PI}}$ was often undefined, so not estimable and hence its variance was not reliable. This relative bias represents the percentage deviation of the estimator (${\hat{\mathrm{Var}}}_{l}$) from the assumed true population value (noting that 100% means the estimator is twice the true value). We also obtained the coverage of nominal 95% Wald intervals (i.e., using the 0.975th quantile of the standard normal z distribution). Importantly, our pilot simulations showed that the delta‐method variance could blow up when θ and n are small, by as much as $10^{5}-10^{10}$. Therefore, we set an upper limit of 20 for the delta‐method variance (this was never an issue for the SAFE variance, which could only reach values around 10 and never exceeded 20; the frequency with which the delta‐method variance exceeded 20 was recorded).

### 2.4 Simulation Results

**FIGURE 1:**
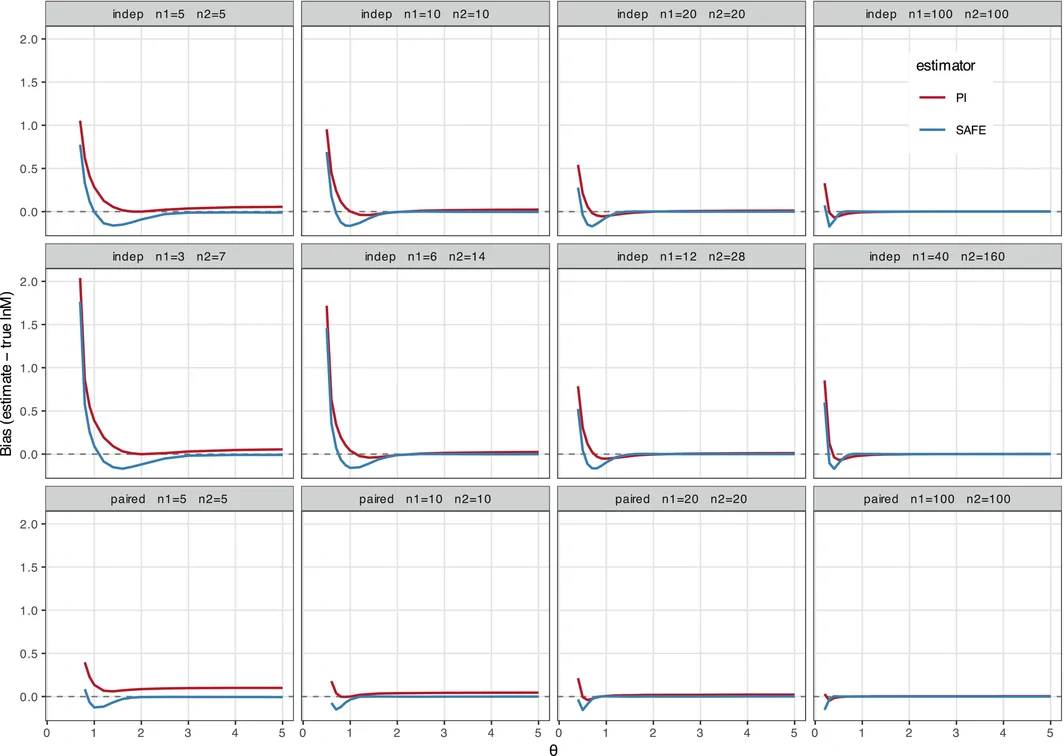
Bias of $\hat{\mathrm{lnM}}$ (estimate minus true value) across all designs in relation to θ, which is the difference between the mean of group 1 $\mu_{1}\left(=0\right)$ and that of group 2 $\mu_{2}\left(=\theta \right)$. Notably, because within‐group standard deviations are fixed at 1 ($\sigma_{1}=\sigma_{2}=1$), our mean difference θ is mathematically equivalent to the true standardised mean difference (d), making it effectively a unit‐free scale here. Solid lines: Plug‐in (PI) estimator (red lines): SAFE estimator (blue lines). Horizontal dashed line denotes zero bias. The facets highlight three design groups: Independent equal (indep with $n_{1}=n_{2}$), independent unequal (indep with $n_{1}\neq n_{2}$), and paired (paired). The y‐axis is shown on the natural logarithm (ln) scale. Note that some values for small sample size scenarios seem to be truncated, as these values could not be defined.

Across all scenarios, the point estimators displayed the same qualitative pattern. When the true difference between the group means was trivial (θ<1) and samples were very small ($n_{i}\leq 10$) both methods (the plug‐in and SAFE) usually overestimated the true lnM; for example, with $n_{1}=n_{2}=5$ (independent) and θ=0.7 the upward bias approached one log‐unit (Figure 1) although SAFE underestimated in some ranges of θ. The bias fell rapidly with either increasing θ or increasing n, becoming negligible (∣bias∣<0.05) once θ≥1 or the minimum sample size contained at least 20 observations. SAFE reduced the bias by around 23.5% relative to the plug‐in (PI) estimator, an advantage that was most pronounced in the extreme lower‐left corners of each panel in Figure 1. Paired designs exhibited the same shape, but with markedly smaller absolute bias because the within‐pair correlation provides additional information: at θ=0.5 and n=5, the bias was approximately half that of the corresponding independent case design.

**FIGURE 2:**
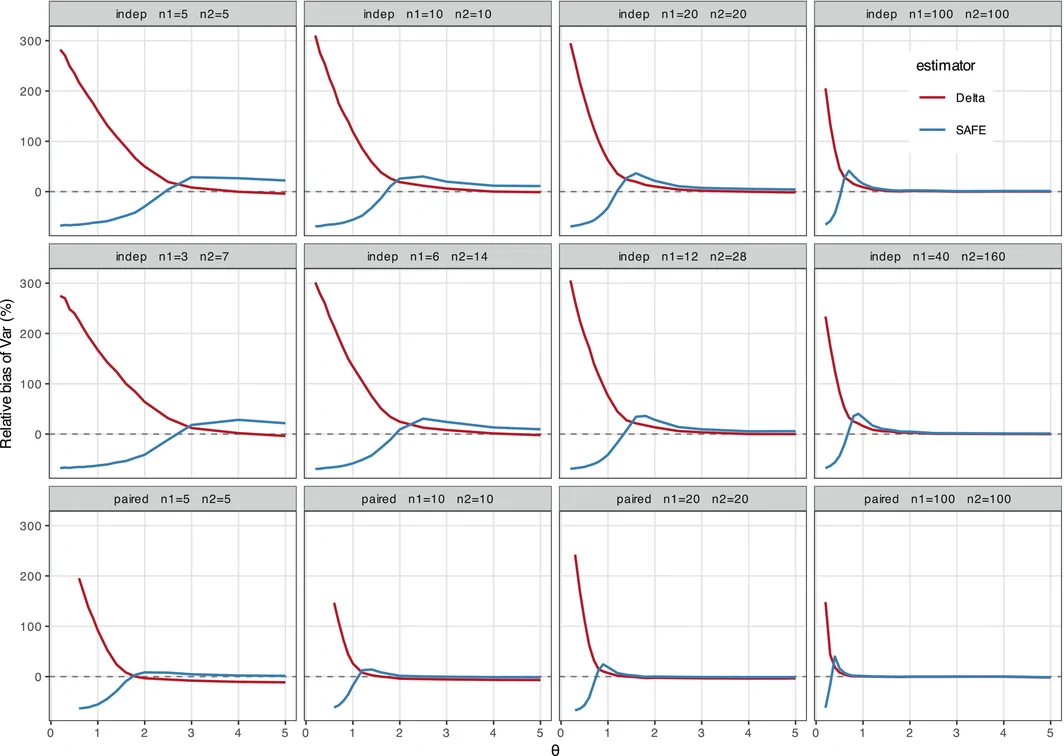
Relative bias of the estimated sampling variance of $\hat{\mathrm{lnM}}$ in relation to θ, which is the difference between the mean of group 1 $\mu_{1}\left(=0\right)$ and that of group 2 $\mu_{2}\left(=\theta \right)$, expressed as $100\;\left[\frac{\hat{{\mathrm{Var}}_{l}}-{\mathrm{Var}}_{\mathrm{MC}}\left(\hat{\mathrm{lnM}}\right)}{{\mathrm{Var}}_{\mathrm{MC}}\left(\hat{\mathrm{lnM}}\right)}\right]$. The red line represents the delta‐method estimator and the blue line represents the SAFE estimator. A value of zero (grey dashed line) indicates an unbiased variance estimator; positive values mean over‐estimation. The facets show three design groups: Independent equal (indep with $n_{1}=n_{2}$), independent unequal (indep with $n_{1}\neq n_{2}$) and paired (paired). Note that some values for small sample size scenarios seem to be truncated, as these values could not be defined.

More striking differences emerged for the estimated sampling variances (Figure 2). For very small samples with θ<1, the delta–method variance was seriously inflated, exceeding the Monte‐Carlo variance by approximately 300% (note this is likely to be a conservative estimate of the inflation as we capped the delta‐method variance to be the maximum of 20 for computation reasons in our simulation). This overestimation decayed smoothly and was practically absent for $n_{i}\geq 20$ or θ≥1.5. By contrast, the SAFE variance was less biased over the whole grid with a tendency to under‐estimate (up to by ~80%) when many bootstrap draws were discarded because $MS_{B}^{*}\leq MS_{W}^{*}$ (note that we always collected $10^{5}$ bootstrap draws regardless of the rate of discarded draws; also see supplemental information for the results of 95% coverage, root mean squared errors [RMSE] and Monte Carlo standard errors [MCSE]).

The frequency with which the analytic formula became undefined (because $MS_{B}\leq MS_{W}$) ranged from 63.5% for the most unbalanced design with $n_{1}=3,n_{2}=7$ to 0% when both groups contained at least 100 observations (Table S1). SAFE always produced an estimate, even though approximately 40%–85% of its bootstrap replicates were discarded in the small‐n designs (i.e., $n_{1}+n_{2}=10$). Also, it is notable that when sample sizes are larger than 40 (i.e., $n_{1}+n_{2}\geq 40$), point estimates and SE are mostly reliable. Given these results, when sample sizes are small or when $MS_{B}\leq MS_{W}$, the SAFE method is strictly required to reduce bias and obtain finite estimates; however, when sample sizes are larger than 40, both point estimates and SEs from the plug‐in method become reliable. In our worked examples below, we primarily used the SAFE estimators to account for the smaller sample sizes present (complementary simulations for ∣d∣, reported in the supplemental information, showed upward bias, especially when sample sizes were small).

### 2.5 Interpretation

Before we apply lnM to example datasets, we provide some guidance on the interpretation of the new effect size. A convenient way to read lnM is through the ratio of the between‐component (mean‐separation) SD to the within‐group SD. By definition,

(20) $$\frac{s_{B}}{s_{W}}=e^{\mathrm{lnM}}$$

Thus lnM=0 corresponds to $s_{B}=s_{W}$, values lnM>0 indicate that the between‐group spread exceeds the typical within‐group SD (e.g., lnM=0.69 implies $s_{B}\approx 2s_{W}$), and lnM<0 indicates that the observed separation is smaller than the within‐group SD. Importantly, testing lnM=0 does not ask “are the group means equal?”, but rather “is the between‐group dispersion at least as large as the within‐group SD?”. A significantly negative lnM, therefore, indicates that, even if the mean difference is non‐zero in a strict sense, it is small compared to within‐group noise.

For interpretability, we believe that it is helpful to map lnM onto another familiar effect size that is also expressed in units of within‐group SD, standardised mean difference, SMD, or d (Hedges 1981). Conveniently, we can define SMD (termed, here, $d_{\mathrm{eq}}$, indicating d‐equivalent) in terms of lnM (see Appendix B for derivation):

(21) $$d_{\mathrm{eq}}=\sqrt{\;2e^{2\;\mathrm{lnM}}+\frac{2}{n_{0}}\;}$$

A simple large‐sample approximation (when $2/n_{0}$ is small) is:

(22) $$d_{\mathrm{eq}}\approx \sqrt{2}\;e^{\mathrm{lnM}}=\sqrt{2}\;\frac{s_{B}}{s_{W}}$$

We treat this mapping as an interpretive aid rather than a primary analysis scale; Table 1 gives a simple rule‐of‐thumb correspondence between lnM, $s_{B}/s_{W}$, and the approximate $d_{\mathrm{eq}}$. As mentioned earlier, lnM=0 is surprisingly large in terms of d (d=1.41). Overall average of ∣d∣ should, however, be almost always larger than d for the same dataset, which include both positive and negative d values, so that thinking lnM in terms of d might be misleading if not interpreted carefully (i.e., a relatively large value of $d_{\mathrm{eq}}$ is expected for overall means for a meta‐analysis of magnitude). Bearing this in mind, one practical use of this conversion is to facilitate direct comparison with the large literature of studies and meta‐analyses that report effects as standardised mean differences, such as Cohen's d or Hedges' g. We emphasise that $d_{\mathrm{eq}}$ is a back‐transformed interpretation of lnM, intended to aid comparison with the familiar standardised mean difference literature; it is not itself the primary analysis scale, nor is it identical to the meta‐analytic mean of ∣d∣. Notably, it is possible to centre lnM at a specific SMD value (e.g., d=0.2 as 0 for adjusted lnM; see Appendix C for a full exploration). Yet we will not use the adjusted version of lnM because it is more informative to compare differences in lnM between groups (i.e., contrasts) and slopes of lnM rather than interpreting an overall lnM of a dataset, as we show in our examples below. We also note that, given this relationship between lnM and SMD, one might wonder why we do not use ln∣d∣ as an effect size. In Appendix D, we explain the limitations of ln∣d∣ and our preference in the use of lnM.

**TABLE 1:** Rule‐of‐thumb mapping from lnM to the ratio $s_{B}/s_{W}=e^{\mathrm{lnM}}$ and the approximate absolute standardised mean difference $d_{\mathrm{eq}}\approx \sqrt{2}\;e^{\mathrm{lnM}}$ (large‐sample approximation); these conversions are useful for meta‐analytic means, which are based on large sample sizes.

lnM	$s_{B}/s_{W}=e^{\mathrm{lnM}}$	$d_{\mathrm{eq}}\approx \sqrt{2}\;e^{\mathrm{lnM}}$
−2.0	≈0.14	≈0.19
−1.5	≈0.22	≈0.32
−1.0	≈0.37	≈0.52
−0.7	≈0.50	≈0.70
−0.4	≈0.67	≈0.95
0.0	1.00	≈1.41

## 3 Worked Examples

### 3.1 Meta‐Analytic Models Considered

In the two worked examples below, we reanalyse published datasets whose original studies used ∣d∣. In both cases, we use multilevel meta‐analysis and meta‐regression models to account for dependence among effect sizes within studies (e.g., multiple effect‐size estimates per study). Let $y_{\mathrm{ij}}$ denote the ith effect size from study j, with corresponding known sampling variance $v_{\mathrm{ij}}$. We first fit an intercept‐only three‐level meta‐analytic model (Nakagawa and Santos 2012; Cheung 2014):

(23) $$y_{\mathrm{ij}}=\beta_{0}+u_{0j}+w_{\mathrm{ij}}+\varepsilon_{\mathrm{ij}}$$

where $\beta_{0}$ is the overall mean effect, $u_{0j}∼N\left(0,\sigma_{\text{study}}^{2}\right)$ is the random intercept at the study level, $w_{\mathrm{ij}}∼N\left(0,\sigma_{\text{effect}}^{2}\right)$ is an additional random effect at within‐study level (i.e., effect‐size level), and $\varepsilon_{\mathrm{ij}}∼N\left(0,v_{\mathrm{ij}}\right)$ is the sampling error (although we have two levels: between‐ and within‐study level, the model is referred to “three level” in the literature, considering the sampling error random effect as another level). Between‐study variance is denoted by $\sigma_{\text{study}}^{2}$ and within‐study (among‐effect) variance by $\sigma_{\text{effect}}^{2}$ along with effect‐size specific sampling variance $v_{\mathrm{ij}}$. Notably, this type of meta‐analysis is called the inverse‐variance method because effect sizes are weighted by the inverse of their sampling variances (Borenstein et al. 2021). Given our simulation results, we also use a subset of the data containing only effect sizes based on sample sizes larger than 40 (i.e., $n_{1}+n_{2}\geq 40$) to run a sensitivity analysis. Such an approach helps prevent overestimating the meta‐analytic mean cf. Ioannidis et al. (2017). To examine how effect sizes vary with moderators, we extend Equation (23) to a three‐level meta‐regression model:

(24) $$y_{\mathrm{ij}}=\beta_{0}+\sum_{p=1}^{P}\beta_{p}x_{p,\mathrm{ij}}+u_{0j}+w_{\mathrm{ij}}+\varepsilon_{\mathrm{ij}}$$

where $x_{p,\mathrm{ij}}$ is the value of the pth moderator (p=1,…,P) for effect size i in study j, and $\beta_{p}$ is the corresponding meta‐regression coefficient.

To assess small‐study effects (the tendency for smaller studies to report larger effects), we use conversions of the harmonic‐mean sample size ($1/\sqrt{{\tilde{n}}_{0}}$ and $1/{\tilde{n}}_{0}$ when ${\tilde{n}}_{0}=1/2n_{0}$ with the harmonic‐mean sample size defined as above) as a moderator, following the approach proposed by Nakagawa et al. (2022), a modified multilevel version of the original Egger's regression test (Egger et al. 1997). We then fit a small‐study effect model of the form:

(25) $$y_{\mathrm{ij}}=\beta_{0}+\beta_{1}\frac{1}{\sqrt{{\tilde{n}}_{0\mathrm{ij}}}}+u_{0j}+w_{\mathrm{ij}}+\varepsilon_{\mathrm{ij}}$$

where $\beta_{1}$ quantifies the association between lnM and the half harmonic‐mean sample size. A significantly negative or positive $\beta_{1}$ would indicate that smaller studies (lower $n_{0\mathrm{ij}}$) tend to yield larger effect size values, consistent with small‐study effects (Jennions and Møller 2002; Sterne and Egger 2001). Also, once we find $\beta_{1}\neq 0$, we fit the model with $1/{\tilde{n}}_{0\mathrm{ij}}$ instead of $\sqrt{1/{\tilde{n}}_{0\mathrm{ij}}}$ and its intercept $\beta_{0}$ as a bias‐corrected overall effect (following Nakagawa et al. 2022) (cf. Stanley and Doucouliagos 2014; Irsova et al. 2025). Although Nakagawa et al. (2022) has recommended including all important moderators for this model to account for heterogeneity due to such moderators, we only run this uni‐moderator small‐study effect test in the examples below. Importantly, we should never use sampling SE in place of $\sqrt{1/{\tilde{n}}_{0\mathrm{ij}}}$, because the point estimate and sampling SE are strongly correlated in lnM as discussed above.

Finally, it is important to emphasise that the example datasets are used here primarily to illustrate the use of lnM, rather than to reproduce the original authors' inferential goals. Accordingly, our model structures and choice of moderators differ from those in the original analyses (e.g., we do not include phylogenetic random effects) (cf. Cinar et al. 2022; Williams et al. 2025); therefore, the numerical results are not directly comparable with those in the original studies. Yet, we report results for both lnM and conventional effect‐size measures (i.e., ∣d∣) so that readers can compare lnM‐based inferences with familiar metrics in each example. We used the R package metafor (Viechtbauer 2010) for model implementation with a helper function to estimate lnM from orchaRd (Nakagawa et al. 2021, 2023). (the code and complete results are presented in https://itchyshin.github.io/meta‐analysis_of_magnitude/).

### 3.2 Example 1: The Effect of Poor Early‐Life Nutrition

**FIGURE 3:**
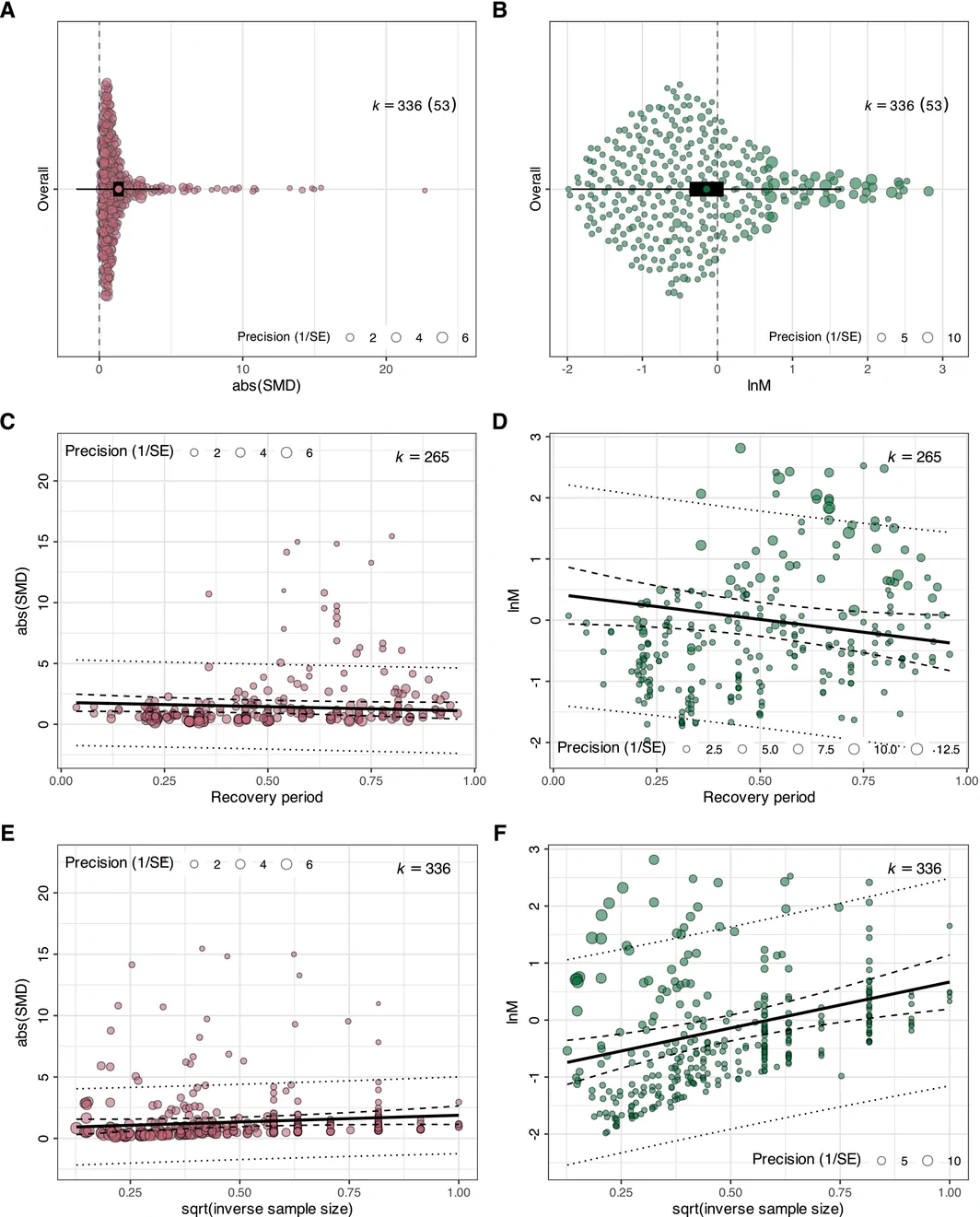
Example 1: The effects of poor early‐life nutrition. (A) Orchard plot for the multilevel meta‐analysis of ∣d∣ (= abs(SMD)) for the 336 “Condition” effect sizes. The y‐axis ‘Overall’ represents the jittered individual effect sizes. (B) Orchard plot for the multilevel meta‐analysis of $\ln M_{\text{SAFE}}$. Coloured points (red for ∣d∣, green for lnM) are study‐level effects, with circle area proportional to precision (1/SE); black circles give meta‐analytic means, thick horizontal lines show 95% confidence intervals, and thin dotted lines show 95% prediction intervals. (C) Bubble plot for meta‐regression on Recovery days for ∣d∣. (D) Bubble plot for meta‐regression on Recovery days for lnM. Point size is proportional to precision, solid lines show fitted slopes, dashed lines show 95% confidence limits and outer dotted lines show 95% prediction limits. (E) Egger‐type regression of ∣d∣ on $\sqrt{1/{\tilde{n}}_{0\mathrm{ij}}}$ (= sqrt(inverse sample size)). (F) Egger‐type regression of lnM on $\sqrt{1/{\tilde{n}}_{0\mathrm{ij}}}$, using the same plotting conventions for regression line, confidence limits and prediction limits; k is the number of effect sizes, while the number in parentheses is the number of studies.

We re‐analysed 683 control‐treatment pairs from Almeida et al. (2021), who investigated early nutritional impacts on a broad taxonomic range of animals (vertebrates and invertebrates), computing ∣d∣ and lnM for each comparison. The delta‐method estimator for lnM failed for 270 entries, whereas the SAFE procedure returned finite point estimates and variances for all 683 entries, rescuing almost 40% of the dataset for variance‐shift analysis. A multilevel meta‐analysis of the dataset, whose outcome was on “Condition” with 336 effect sizes (Figure 3A,B) gave a large overall mean ∣d∣ ($\beta_{0}^{|d|}=1.35$, 95% CI: 0.98–1.71 with $I_{\text{total}}^{2}=95.45\%$). The overall effect ${\mathrm{lnM}}_{\text{SAFE}}$ was negative and non‐significant ($\beta_{0}^{\mathrm{lnM}}=-0.14$, 95% CI: −0.37 to 0.08 with $I_{\text{total}}^{2}=89.43\%$). Yet, the conversion $d_{\mathrm{eq}}$ showed a similar result with ∣d∣ ($\beta_{0}^{d_{\mathrm{eq}}}=1.22$, 95% CI: 0.98–1.54), as expected.

Next, we ran a meta‐regression with three continuous moderators (with 265 effect sizes): (1) severity of nutritional deprivation, (2) duration of deprivation and (3) recovery period. One notable result is that for ∣d∣, the slope for the recovery period was negative but not significantly so ($\beta_{\text{recovery}}^{|d|}=-0.70$, 95% CI: −1.69 to 0.28), whereas for lnM, the slope was significantly negative ($\beta_{\text{recovery}}^{\mathrm{lnM}}=-0.84$, 95% CI: −1.61 to −0.06), implying that as the recovery period increases, the two groups become more similar; the nutritionally deprived group was able to recover (Figure 3C,D; the other variables were statistically significant for both ∣d∣ and lnM). This difference in inference is mainly due to the boundary at zero for ∣d∣.

Egger‐type regressions (small‐study‐effect text) (Figure 3E,F) suggested a weak and non‐significant small‐study pattern for ∣d∣ ($\beta_{\mathrm{nSE}}^{|d|}=1.09$, 95% CI: −0.20 to 2.37) but a stronger and significant pattern for lnM ($\beta_{\mathrm{nSE}}^{\ln M}=1.61$, 95% CI: 0.79–2.44). Therefore, we estimated a bias‐corrected overall effect ($\beta_{0}^{{\mathrm{lnM}}_{\text{SAFE}}}=-0.59$, 95% CI: −0.91 to −0.27; $\beta_{0}^{d_{\mathrm{eq}}}=0.78$, 95% CI: 0.57–1.08); this represents around 65% reduction in the overall mean estimation (in terms of $d_{\mathrm{eq}}$). Similarly, our sensitivity analysis by restricting effect sizes based on $n_{1}+n_{2}\geq 40$ showed a smaller overall effect than the original overall estimate ($\beta_{0}^{{\mathrm{lnM}}_{\text{SAFE}}}=-0.41$, 95% CI: −0.95 to 0.12; $\beta_{0}^{d_{\mathrm{eq}}}=0.94$, 95% CI: 0.55–1.60). Therefore, both of the above‐mentioned overall means are likely to be severely overestimated.

### 3.3 Example 2: The Impacts of Anthropogenic Noise

**FIGURE 4:**
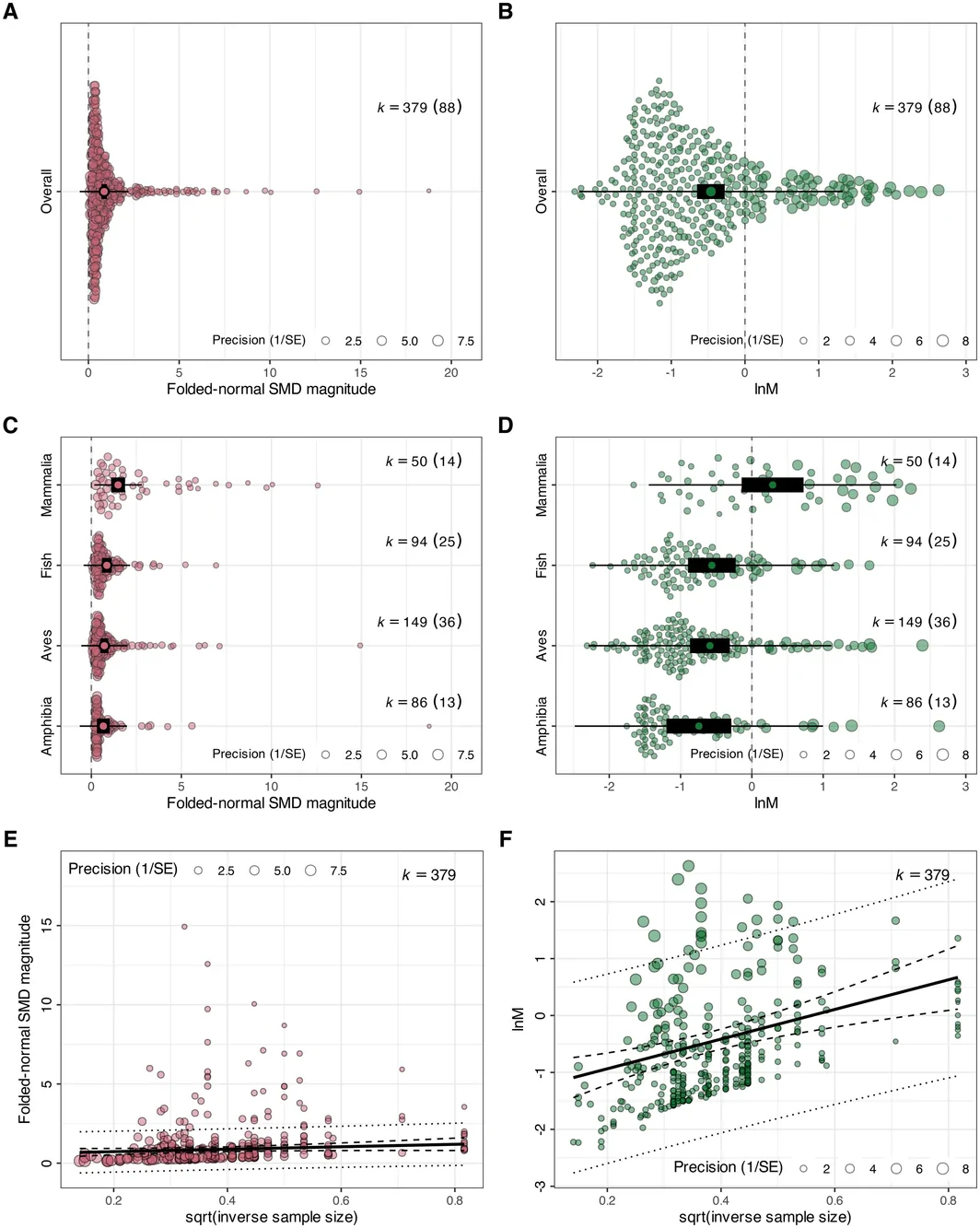
Example 2: The impacts of anthropogenic noise. (A) Orchard plot for the multilevel meta‐analysis of ∣d∣ (= abs(SMD)) across 379 effect sizes from 88 studies. The y‐axis ‘Overall’ represents the jittered individual effect sizes. (B) Orchard plot for the multilevel meta‐analysis of $\ln M_{\text{SAFE}}$. Symbols and lines are as in Figure 3A,B, with coloured points (red for ∣d∣, green for lnM). (C) Orchard plot from the taxon‐specific meta‐regression, showing estimated ∣d∣ for amphibians, birds, fishes and mammals. (D) Orchard plot from the taxon‐specific meta‐regression for lnM with 95% confidence and prediction intervals; circle size again reflects precision. (E) Egger‐type regression of ∣d∣ on $\sqrt{1/{\tilde{n}}_{0\mathrm{ij}}}$ (= sqrt(inverse sample size)). (F) Egger‐type regression of lnM on $\sqrt{1/{\tilde{n}}_{0\mathrm{ij}}}$, with solid lines for fitted slopes, dashed lines for 95% confidence limits, and outer dotted lines for 95% prediction limits; k is the number of effect sizes while the number in parentheses are the number of studies.

For the dataset of Kunc and Schmidt (2019), which explored the impact of anthropogenic noise on animals, with 486 entries (effect sizes); the delta‐method lnM failed for 177 of these, whereas SAFE produced lnM estimates and variances for all contrasts, rescuing ~36% of the dataset. After restricting the dataset to birds, mammals, amphibians and fishes (379 effect sizes from 88 studies), a multilevel meta‐analysis (Figure 4A,B) showed a moderate‐to‐large mean shift ($\beta_{0}^{|d|}=0.87$, 95% CI: 0.71–1.02 with $I_{\text{total}}^{2}=84.99\%$); lnM showed a negative overall effect, indicating the separation was small relative to within‐group noise ($\beta_{0}^{\mathrm{lnM}}=-0.46$, 95% CI: −0.65 to −0.27 with $I_{\text{total}}^{2}=83.57\%$). Yet, both results are consistent with each other when comparing ∣d∣ and $d_{\mathrm{eq}}$ ($\beta_{0}^{d_{\mathrm{eq}}}=0.89$, 95% CI: 0.74–1.07).

Using taxon as a moderator (a categorical moderator with 4 levels; Figure 4C,D), ∣d∣‐based meta‐regression suggested all four taxa respond positively to noise, with mammals showing the largest mean shift ($\beta_{\text{mammalia}}^{|d|}=1.48$, 95% CI: 1.10–1.87). The corresponding ${\mathrm{lnM}}_{\text{SAFE}}$ model gave negative coefficients for amphibians, birds and fishes (e.g., $\beta_{\text{amphibia}}^{\mathrm{lnM}}=-0.74$, 95% CI: −1.20 to −0.29), but a near‐zero coefficient for mammals ($\beta_{\text{mammalia}}^{\mathrm{lnM}}=0.29$, 95% CI: −0.14 to 0.73). Yet, these results are consistent across contrasts among taxa; for both ∣d∣ and lnM, only mammals are significantly and positively different from the other taxa.

Egger‐type regressions again produced a weakly positive yet non‐significant pattern for ∣d∣ ($\beta_{1}^{|d|}=0.77$, 95% CI: −0.07 to 1.61) but a strong and significant association for lnM ($\beta_{1}^{\mathrm{lnM}}=2.60$, 95% CI: 1.40–3.82; Figure 4E,F). Our bias‐corrected estimate suggests the “true” overall mean might be approximately 38% smaller than the original estimate ($\beta_{0}^{\mathrm{lnM}}=-0.93$, 95% CI: −1.22 to −0.64; $\beta_{0}^{d_{\mathrm{eq}}}=0.56$, 95% CI: 0.42–0.74). Also, our sensitivity analysis for the overall effect revealed a smaller overall effect than the original overall estimate, but more similar to the bias‐corrected estimate ($\beta_{0}^{\mathrm{lnM}}=-0.76$, 95% CI: −1.06 to −0.46; $\beta_{0}^{d_{\mathrm{eq}}}=0.66$, 95% CI: 0.49–0.89). Therefore, all the meta‐analytic results above are likely inflated, although contrasts (differences between taxonomic groups) would remain valid. Fortunately, using lnM allows adjustment for small‐study effects (see the online supplements; see the Discussion section for more details).

## 4 Discussion

We have introduced lnM, which quantifies the magnitude of separation between two groups as the ratio of between‐group to within‐group standard deviations, derived directly from the components of one‐way ANOVA. By targeting “how far apart” groups are, irrespective of direction, and working on the log scale, lnM avoids the systematic bias that arises when absolute‐valued effect sizes (e.g., ∣lnRR∣ or ∣SMD∣) are analysed directly (Morrissey 2016a, 2016b), while remaining compatible with standard random‐effects (multilevel) meta‐analysis and meta‐regression (Hedges 1981; Hedges et al. 1999; Viechtbauer 2010; Cheung 2014). Conceptually, lnM complements existing log‐ratio metrics such as lnRR, lnVR and lnCVR (Nakagawa et al. 2015; Senior et al. 2020), but targets a different quantity: the magnitude of mean separation relative to within‐group noise. The zero point is interpretable: lnM = 0 corresponds to between‐group dispersion matching the within‐group SD, so inference is framed in terms of whether between‐group spread meets or exceeds within‐group variability, not whether group means are identical. Because it is constructed from means and standard deviations rather than raw ratios of means, lnM can be applied to both ratio‐ and interval‐scale traits, including outcomes where lnRR is undefined or unstable (e.g., means near zero or with mixed signs). In this sense, lnM offers a pragmatic route to unsigned magnitude synthesis that complements the treatments of ∣d∣ and $d^{2}$ based on folded‐t and non‐central‐F distributions, proposed by Kulinskaya and Hoaglin (2023).

Our simulation study clarifies where lnM is reliable and where it becomes fragile. When the true separation is trivial and sample sizes are very small, plug‐in lnM tends to be upwardly biased and the first‐order delta‐method sampling variance can be severely inflated, reflecting the algebraic sensitivity of lnM as the between‐group mean square $MS_{B}$ approaches the within‐group mean square $MS_{W}$. This inflation leads to very large standard errors, which are simply not usable in meta‐analysis. As mean differences or sample sizes increase, both bias and variance distortion decay rapidly, and plug‐in lnM with delta‐method variances performs well, consistent with earlier work on delta‐method effect sizes such as lnRR, lnVR and lnCVR (Lajeunesse 2011, 2015; Senior et al. 2020). The SAFE bootstrap (Mandel 2013; Fletcher and Jowett 2022; Nakagawa et al. 2026) reduces point‐estimate bias across the parameter grid and stabilises variance estimates, especially in the small‐n, small‐separation region, and crucially continues to return finite estimates when $MS_{B}\leq MS_{W}$, where the analytic definition of lnM fails. In practice, this means that SAFE allows analysts to retain all entries with small separations that would otherwise be discarded, while providing more realistic standard errors (although they are downwardly biased for small separations and sample sizes; Figure 2) for routine meta‐analysis and meta‐regression.

The worked examples demonstrate how lnM behaves for real datasets and how it compares with the more familiar ∣d∣. In the early‐life nutrition data (Almeida et al. 2021), SAFE‐based lnM estimates allowed us to retain all entries where $MS_{B}\leq MS_{W}$ and revealed moderator patterns, such as the decline in separation with increasing recovery time, that were less apparent on the ∣d∣ scale. In the anthropogenic noise dataset (Kunc and Schmidt 2019), lnM and ∣d∣ agreed qualitatively on overall magnitudes and taxon‐level differences, yet lnM made small‐study (publication‐bias) patterns clearer with Egger‐type regressions using ${\tilde{n}}_{0}$‐based moderators (Egger et al. 1997; Nakagawa et al. 2022). This showed more pronounced small‐study effects for lnM, and bias‐adjusted lnM means translated into noticeably smaller d‐equivalents than naive summaries. In both examples, lnM could be analysed with standard multilevel models, and the back‐transforms to $d_{\mathrm{eq}}$ provided familiar interpretations.

The proposed lnM has limitations that warrant caution in certain circumstances. When separations and sample sizes are both very small, all estimators of lnM are noisy and biased to some degree (Figure 1), and SAFE can only mitigate, not remove, this problem. In such cases, it may be more honest to conclude that dispersion differences are small and imprecisely estimated; especially if overall estimates are small, they are likely overestimates. Yet, our proposed sensitivity analysis, with effect sizes satisfying $n_{1}+n_{2}\geq 40$, should largely eliminate this issue. In addition, the structural dependence of lnM's variance on ($MS_{B}-MS_{W}$) means that larger lnM values tend to receive higher precision and greater weight, which makes this effect size highly sensitive to publication bias. That is, a small‐study effect could bias the overall estimate more than other effect size statistics (Egger et al. 1997; Sterne and Egger 2001). Note that this relationship itself does not bias estimates in meta‐analysis, and the issue only arises with publication bias. Yet, this tendency due to publication bias (small‐study effect) could be substantially mitigated by utilising the harmonic mean of sample sizes. That is, using ${\tilde{n}}_{0}$ rather than standard errors (see Nakagawa et al. 2022).

**FIGURE 5:**
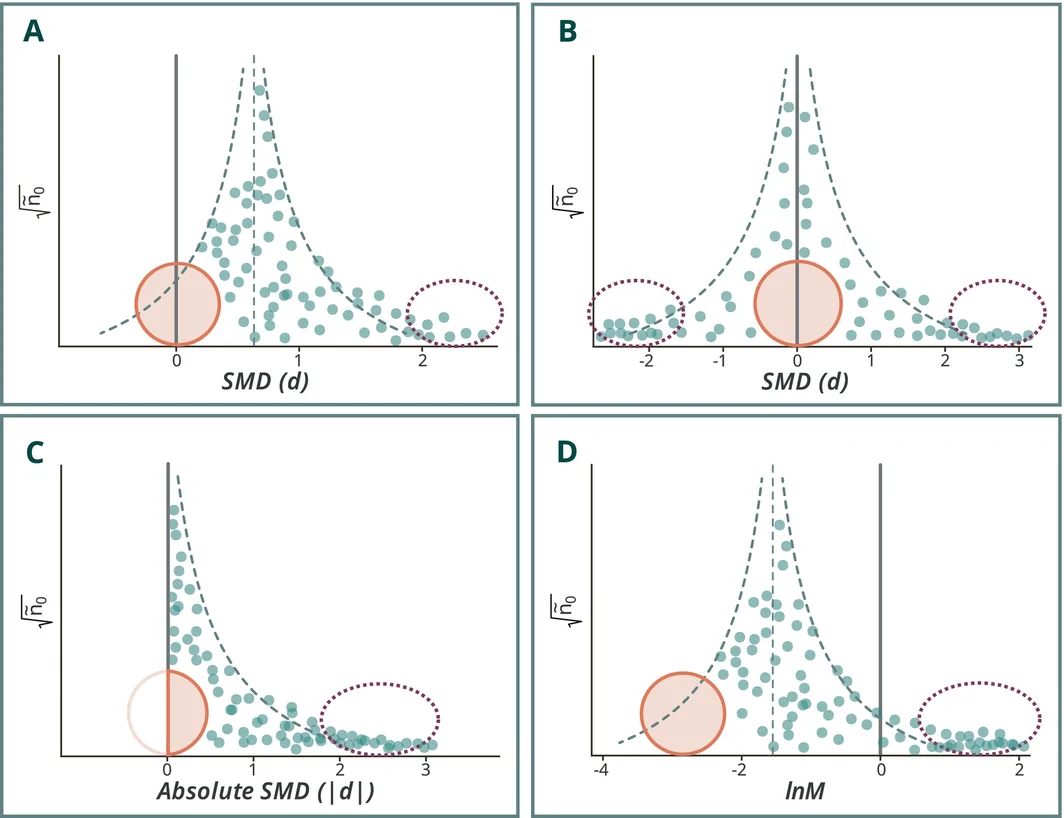
Funnel plots illustrate how magnitude transformations can hide or reveal small‐study effects. The vertical axis represents effect‐size precision, shown as $\sqrt{{\tilde{n}}_{0}}$, where ${\tilde{n}}_{0}=n_{0}/2$ and $n_{0}=2n_{1}n_{2}/\left(n_{1}+n_{2}\right)$ is the harmonic‐mean sample size for a two‐group comparison. Teal points are individual effect‐size estimates; dashed curves are pseudo‐funnel limits (illustrative 95% bounds) that narrow with increasing precision (sample size); note true funnel limits can only be calculated with precision (1/SE) not with $\sqrt{{\tilde{n}}_{0}}$. Solid vertical lines indicate the null value (0 on each effect‐size scale). The orange circle highlights the region of small, imprecise studies (effect sizes) with near‐null estimates that would be expected under no selection; the dotted purple ellipses highlight an excess of extreme estimates among low‐precision (low‐n) studies. (A) A conventional funnel plot for a signed standardised mean difference, $\mathrm{SMD}\left(d\right)$, showing the familiar funnel asymmetry associated with small‐study effects (often interpreted as publication bias or selective reporting that favours larger effects in smaller studies). (B–D) The same underlying dataset (note that the dataset for A is different from that of B–D), shown on three analytically related scales: (B) signed $\mathrm{SMD}\left(d\right)$ centred at 0, where two‐sided selection for statistical significance can yield a symmetric “missing‐centre” pattern (few small studies near d≈0) that does not produce the classic left–right funnel asymmetry targeted by Egger‐type regressions; (C) absolute standardised mean differences, ∣d∣, which folds (B) at 0 and induces a hard boundary and strong skew, violating the linear/Gaussian assumptions underpinning Egger‐type regressions and making a funnel plot inherently non‐symmetric; (D) the ln‐magnitude effect size, lnM, an unbounded and near‐Gaussian transformation of magnitude that re‐expresses small separations as negative values and large separations as positive values, thereby reinstating a funnel‐asymmetry pattern that can be assessed using Egger‐type regression with a sample‐size‐based term (e.g., $1/\sqrt{{\tilde{n}}_{0}}$) in the same spirit as panel (A).

Relatedly, with ${\tilde{n}}_{0}$, lnM allows us not only to test for but also potentially to correct publication bias due to the small‐study effect (for extensions of publication‐bias tests, see Nakagawa et al. 2025). Figure 5 depicts how lnM could identify a small‐study effect (Figure 5D) even when it is not feasible to detect the small study effects using signed effect sizes such as d and lnRR (Figure 5A,B; see also the online tutorial for how this ability of lnM could be used to correct for estimation bias in meta‐analysis and meta‐regression models). Notably, Egger‐type regression cannot be applied appropriately to absolute effect sizes such as ∣d∣ and ∣lnRR∣ due to the zero boundary (Figure 5C). This could mean that, as we saw in our worked examples, past meta‐analyses of magnitudes using ∣d∣ and ∣lnRR∣ are likely to have produced severely overinflated results due to publication bias, which has never been discussed in the literature as far as we know (cf. Morrissey 2016a, 2016b). This presents an excellent opportunity for future research to reanalyse past meta‐analyses of magnitudes using lnM to check and correct for publication bias, which could yield novel insights and refine previous conclusions. Therefore, the above‐mentioned limitations of lnM, we believe, appear to be outweighed by the benefits of lnM over absolute‐valued effect sizes. We also hope that future methodological research will identify improved estimators of lnM and its sample variance to address the current limitations, as we have so far been unable to identify such estimators.

Taken together, despite its limitations, lnM offers a simple, meta‐regression‐friendly way to quantify and synthesise magnitudes of separation between groups, avoiding the pathologies of absolute‐value effect sizes while remaining compatible with flexible multilevel and meta‐regression models (Nakagawa and Santos 2012; Cheung 2014). Combined with the SAFE bootstrap, routine sensitivity analyses and the use of ${\tilde{n}}_{0}$‐based moderators, lnM provides a practical tool for asking how far populations, treatments or strategies sit from each other, in the spirit of Lynch's variance‐ratio framework (Lynch 1990) and more recent work on variation‐focused effect sizes (Nakagawa et al. 2015; Senior et al. 2020). We hope lnM will encourage more ecologists, evolutionary biologists and other scientists to treat magnitude as a main target of synthesis, alongside mean shifts and variance ratios, thereby sharpening inference about the strength and structure of ecological and evolutionary processes and beyond.

## Author Contributions

Conceptualization: S.N., R.E.O. Data curation: S.N. Formal analysis: S.N., R.E.O. Funding acquisition: S.N. Investigation: S.N. Methodology: S.N. Project administration: S.N., S.O. Supervision: S.N. Validation: S.O., E.L., D.W.A.N. Visualisation: A.M., S.N. Writing – original draft: S.N. Writing – review and editing: All authors.

## Funding

S.N., A.M., E.L. and S.O. were supported by the Canada Excellence Research Chair (CERC‐2022‐00074) and NSERC Discovery Grant (RGPIN‐2025‐04813). Also, S.N. and M.L. were supported by the Australian Research Council Discovery Grant (DP230101248). D.W.A.N. and A.M.S. were supported by an ARC Future Fellowship (FT220100276; FT230100240).

## Conflicts of Interest

The authors declare no conflicts of interest.

## Data Availability

All data, scripts and relevant files used for this study can be found at the GitHub repository (https://github.com/itchyshin/meta‐analysis_of_magnitude) and a version of it is archived at Zenodo (https://zenodo.org/records/20449552) once it is accepted for publication.

## Appendix A Delta‐Method Derivation of the Sampling Variance of lnM

In this appendix, we outline how the sampling variances in Equations (5) and (6) follow from a first‐order Taylor expansion (delta method) applied to lnM. We treat the two‐group case with independent samples first and then the paired (dependent) design.

### 1 Independent Groups

Recall that for two independent groups with sample sizes $n_{1},n_{2}$, means ${\bar{X}}_{1},{\bar{X}}_{2}$, variances $s_{1}^{2},s_{2}^{2}$, and harmonic‐mean sample size $n_{0}=2n_{1}n_{2}/\left(n_{1}+n_{2}\right)$, the ANOVA mean squares are:

(A1)
$$MS_{B}=\frac{n_{1}n_{2}}{n_{1}+n_{2}}\;{\left({\bar{X}}_{1}-{\bar{X}}_{2}\right)}^{2}=\frac{n_{1}n_{2}}{n_{1}+n_{2}}\;\delta^{2}$$

(A2)
$$MS_{W}=\frac{\left(n_{1}-1\right)s_{1}^{2}+\left(n_{2}-1\right)s_{2}^{2}}{n_{1}+n_{2}-2}$$

with $\delta ={\bar{X}}_{1}-{\bar{X}}_{2}$. We also define the difference:

(A3)
$$\Delta =MS_{B}-MS_{W}$$

and note that the pooled within‐group variance is $s_{W}^{2}=MS_{W}$. The log‐magnitude effect size is:

(A4)
$$\mathrm{lnM}=\frac{1}{2}\left[\ln\left(\Delta \right)-\ln\left(n_{0}\right)-\ln\left(MS_{W}\right)\right]$$

For the independent design, the random quantities entering: Equation (A4) are $\left({\bar{X}}_{1},{\bar{X}}_{2},s_{1}^{2},s_{2}^{2}\right)$, with:

(A5)
$$\begin{array}{c} \mathrm{Var}\left({\bar{X}}_{1}\right)=\frac{s_{1}^{2}}{n_{1}},\;\mathrm{Var}\left({\bar{X}}_{2}\right)=\frac{s_{2}^{2}}{n_{2}},\;\mathrm{Cov}\left({\bar{X}}_{1},{\bar{X}}_{2}\right)=0 \end{array}$$

(A6)
$$\begin{array}{c} \mathrm{Var}\left(s_{1}^{2}\right)=\frac{2s_{1}^{4}}{n_{1}-1},\;\mathrm{Var}\left(s_{2}^{2}\right)=\frac{2s_{2}^{4}}{n_{2}-1},\;\mathrm{Cov}\left(s_{1}^{2},s_{2}^{2}\right)=0 \end{array}$$

and, under normal‐theory assumptions:

(A7)
$$\mathrm{Cov}\left({\bar{X}}_{j},s_{k}^{2}\right)=0\;\text{for}\;\mathrm{all}\;\;j,k\in \left\{1,2\right\}$$

We now treat lnM as a function of the vector:

$$\zeta ={\left({\bar{X}}_{1},{\bar{X}}_{2},s_{1}^{2},s_{2}^{2}\right)}^{⊤},$$

and apply the delta method:

(A8)
$${\mathrm{Var}}_{\mathrm{IND}}\left(\mathrm{lnM}\right)\approx \nabla_{\zeta }{\mathrm{lnM}}^{⊤}\;\Sigma_{\zeta }\;\nabla_{\zeta }\mathrm{lnM}$$

where $\Sigma_{\zeta }$ is the covariance matrix of ζ and $\nabla_{\zeta }\mathrm{lnM}$ is the gradient of Equation (A4) with respect to $\left({\bar{X}}_{1},{\bar{X}}_{2},s_{1}^{2},s_{2}^{2}\right)$.

To simplify notation, note that:

(A9)
$$\delta ={\bar{X}}_{1}-{\bar{X}}_{2},\;\;s_{D}^{2}=\frac{s_{1}^{2}}{n_{1}}+\frac{s_{2}^{2}}{n_{2}}$$

so that $s_{D}^{2}$ is the sampling variance of δ under independence. Differentiating Equation (A4) and using the chain rule for $\Delta =MS_{B}-MS_{W}$ yields:

(A10)
$$\frac{\partial \;\mathrm{lnM}}{\partial {\bar{X}}_{1}}=\frac{1}{2}\left(\frac{1}{\Delta }\;\frac{\partial \Delta }{\partial {\bar{X}}_{1}}\right)=\frac{1}{2}\left(\frac{1}{\Delta }\;\frac{2n_{1}n_{2}}{n_{1}+n_{2}}\;\delta \right)=\frac{n_{0}}{2}\;\frac{\delta }{\Delta }$$

(A11)
$$\frac{\partial \;\mathrm{lnM}}{\partial {\bar{X}}_{2}}=-\frac{n_{0}}{2}\;\frac{\delta }{\Delta }$$

Similarly, taking derivatives with respect to $s_{1}^{2}$ and $s_{2}^{2}$ and collecting terms shows that all contributions from the mean squares can be written in terms of Δ, $MS_{W}$ and $MS_{B}$. After substituting the variances and covariances of $\left({\bar{X}}_{1},{\bar{X}}_{2},s_{1}^{2},s_{2}^{2}\right)$ and simplifying, we obtain:

(A12)
$${\mathrm{Var}}_{\mathrm{IND}}\left(\mathrm{lnM}\right)=\frac{1}{4\Delta^{2}}\left[{\left(\frac{n_{0}}{2}\right)}^{2}\left(2\;s_{D}^{4}+4\;s_{D}^{2}\;\delta^{2}\right)+\frac{MS_{B}^{2}}{MS_{W}^{2}}\;\frac{2\;MS_{W}^{2}}{\;n_{1}+n_{2}-2\;}\right]$$

which is the expression reported in Equation (5) in the main text. Here, the first term inside the brackets arises from the uncertainty in the mean difference δ (via $s_{D}^{2}$), and the second term arises from the sampling variability of $MS_{W}$.

### 2 Paired (Dependent) Groups

For paired data with $n_{1}=n_{2}=n$, means ${\bar{X}}_{1},{\bar{X}}_{2}$, variances $s_{1}^{2},s_{2}^{2}$, and within‐pair correlation r, the between‐ and within‐group mean squares are:

(A13)
$$MS_{B}=\frac{n}{2}\;{\left({\bar{X}}_{1}-{\bar{X}}_{2}\right)}^{2}=\frac{n}{2}\;\delta^{2}$$

(A14)
$$MS_{W}=\frac{s_{1}^{2}+s_{2}^{2}}{2}$$

again with $\delta ={\bar{X}}_{1}-{\bar{X}}_{2}$ and $\Delta =MS_{B}-MS_{W}$. The lnM definition Equation (A4) remains the same, but the joint sampling distribution of $\left({\bar{X}}_{1},{\bar{X}}_{2},s_{1}^{2},s_{2}^{2}\right)$ now includes non‐zero covariances:

(A15)
$$\begin{array}{c} \mathrm{Var}\left({\bar{X}}_{1}\right)=\frac{s_{1}^{2}}{n},\;\mathrm{Var}\left({\bar{X}}_{2}\right)=\frac{s_{2}^{2}}{n},\;\mathrm{Cov}\left({\bar{X}}_{1},{\bar{X}}_{2}\right)=\frac{r\;s_{1}s_{2}}{n} \end{array}$$

(A16)
$$\begin{array}{c} \mathrm{Var}\left(s_{1}^{2}\right)=\frac{2s_{1}^{4}}{n-1},\;\mathrm{Var}\left(s_{2}^{2}\right)=\frac{2s_{2}^{4}}{n-1},\;\mathrm{Cov}\left(s_{1}^{2},s_{2}^{2}\right)=\frac{2r^{2}s_{1}^{2}s_{2}^{2}}{n-1} \end{array}$$

while the covariances between means and variances remain zero under normality:

(A17)
$$\mathrm{Cov}\left({\bar{X}}_{j},s_{k}^{2}\right)=0\;\text{for}\;\mathrm{all}\;\;j,k\in \left\{1,2\right\}$$

The variance of the within‐pair difference $D=X_{1}-X_{2}$ is:

(A18)
$$s_{D}^{2}=s_{1}^{2}+s_{2}^{2}-2r\;s_{1}s_{2}$$

As in the independent case, we apply the delta method with $\zeta ={\left({\bar{X}}_{1},{\bar{X}}_{2},s_{1}^{2},s_{2}^{2}\right)}^{⊤}$ and Equation (A4). Differentiating Δ and $MS_{W}$ now uses the paired definitions of $MS_{B}$ and $MS_{W}$, but the structure of the gradient is analogous. After substituting the covariance matrix of ζ for the paired design and simplifying, the variance of lnM is:

(A19)
$${\mathrm{Var}}_{\mathrm{DEP}}\left(\mathrm{lnM}\right)=\frac{1}{4\Delta^{2}}\left[{\left(\frac{n}{2}\right)}^{2}\left(\frac{2\;s_{D}^{4}}{n^{2}}+\frac{4\;\delta^{2}\;s_{D}^{2}}{n}\right)+\frac{MS_{B}^{2}}{MS_{W}^{2}}\;\frac{s_{1}^{4}+s_{2}^{4}+2\;r^{2}s_{1}^{2}s_{2}^{2}}{2\;\left(n-1\right)}\right]$$

which matches Equation (6) in the main text. The first bracketed term captures the contribution of uncertainty in the mean difference (through $s_{D}^{2}$), while the second term reflects the sampling variability of $MS_{W}$ and the correlation‐induced coupling between $s_{1}^{2}$ and $s_{2}^{2}$.

In both independent and paired designs, the leading factor $1/\left(4\Delta^{2}\right)$ shows that the sampling variance of lnM grows quickly as $MS_{B}$ approaches $MS_{W}$ (i.e., Δ→0), and shrinks rapidly once $MS_{B}$ pulls away from $MS_{W}$, as discussed in the main text.

## Appendix B From ANOVA Components to the d‐Equivalent for lnM

We first derive the expression for InM in terms of the mean difference δ and the within‐group mean square $MS_{W}$, and then obtain the corresponding absolute standardised mean difference $d_{\mathrm{eq}}$.

For two groups with sample sizes $n_{1},n_{2}$, means ${\bar{X}}_{1},{\bar{X}}_{2}$, and variances $s_{1}^{2},s_{2}^{2}$, the one‐way ANOVA mean squares are:

(A20)
$$MS_{B}=\frac{n_{1}n_{2}}{n_{1}+n_{2}}\;{\left({\bar{X}}_{1}-{\bar{X}}_{2}\right)}^{2}=\frac{n_{1}n_{2}}{n_{1}+n_{2}}\;\delta^{2}$$

(A21)
$$MS_{W}=\frac{\left(n_{1}-1\right)s_{1}^{2}+\left(n_{2}-1\right)s_{2}^{2}}{n_{1}+n_{2}-2}$$

where

$$\delta ={\bar{X}}_{1}-{\bar{X}}_{2}$$

The harmonic‐mean sample size is

(A22)
$$n_{0}=\frac{2n_{1}n_{2}}{n_{1}+n_{2}}$$

Following the main text, we define the between‐group and within‐group variance components as follows:

(A23)
$$s_{B}^{2}=\frac{MS_{B}-MS_{W}}{n_{0}},\;\;s_{W}^{2}=MS_{W}$$

so that the log‐magnitude effect size is:

(A24)
$$\mathrm{lnM}=\ln\left(\frac{s_{B}}{s_{W}}\right)=\frac{1}{2}\left[\ln\left(s_{B}^{2}\right)-\ln\left(s_{W}^{2}\right)\right]$$

Substituting Equation (A23) into Equation (A24) gives:

(A25)
lnM=12lnMSB−MSWn0−lnMSW=12lnMSB−MSW−lnn0−lnMSW

Now use Equations (A20) and (A22) to write

$$MS_{B}=\frac{n_{1}n_{2}}{n_{1}+n_{2}}\;\delta^{2}=\frac{n_{0}}{2}\;\delta^{2}$$

Hence,

(A26)
$$MS_{B}-MS_{W}=\frac{n_{0}}{2}\;\delta^{2}-MS_{W}$$

(A27)
$$\mathrm{lnM}=\frac{1}{2}\left[\ln\left(\frac{n_{0}}{2}\;\delta^{2}-MS_{W}\right)-\ln\left(n_{0}\right)-\ln\left(MS_{W}\right)\right]$$

Dividing the argument of the logarithm by $MS_{W}$ and using $s_{W}^{2}=MS_{W}$ yields:

(A28)
lnM=12lnn02δ2−MSWn0MSW=12lnδ22sW2−1n0

Starting from Equation (A28),

$$\mathrm{lnM}=\frac{1}{2}\ln\left(\frac{\delta^{2}}{2\;s_{W}^{2}}-\frac{1}{n_{0}}\right)$$

we exponentiate and rearrange:

(A29)
$$e^{2\;\mathrm{lnM}}=\frac{\delta^{2}}{2\;s_{W}^{2}}-\frac{1}{n_{0}}$$

(A30)
$$\frac{\delta^{2}}{s_{W}^{2}}=2\;e^{2\;\mathrm{lnM}}+\frac{2}{n_{0}}$$

Defining the implied absolute standardised mean difference as follows:

$$d_{\mathrm{eq}}=\frac{|\;\delta \;|}{s_{W}}$$

we obtain the relationship:

(A31)
$$d_{\mathrm{eq}}=\sqrt{\;2e^{2\;\mathrm{lnM}}+\frac{2}{n_{0}}\;}$$

which matches Equation (21).

For typical meta‐analytic applications, the term $2/n_{0}$ is small, yielding the large‐sample approximation.

(A32)
$$d_{\mathrm{eq}}\approx \sqrt{2}\;e^{\mathrm{lnM}}=\sqrt{2}\;\frac{s_{B}}{s_{W}}$$

which we use for the interpretive values reported in Table 1 and matches Equation (22).

## Appendix C Centering lnM to a Target Absolute SMD

We want to re‐anchor lnM so that zero corresponds to a user‐chosen absolute standardised separation $d_{0}=|\;\delta \;|\;/s_{W}$.

From Appendix B, the absolute SMD implied by lnM is:

(A33)
$$d_{\mathrm{eq}}^{2}=2\;e^{2\mathrm{lnM}}+\frac{2}{n_{0}},\;\;n_{0}=\frac{2n_{1}n_{2}}{n_{1}+n_{2}}$$

Setting $d_{\mathrm{eq}}=d_{0}$ in Equation (A33) gives:

(A34)
e2lnM*=d022−1n0=n0d02−22n0

Taking the logarithm and dividing by 2 on both sides gives:

(A35)
$$\begin{array}{c} {\mathrm{lnM}}^{*}=\frac{1}{2}\ln\left(\frac{n_{0}d_{0}^{2}-2}{2n_{0}}\right) \end{array}$$

Note that existence requires $\left(n_{0}/2\right)\;d_{0}^{2}>1$, typically satisfied in meta‐analytic applications.

Subtracting the anchor yields:

(A36)
lnMd0=lnM−lnM*=lnM+12ln2n0n0d02−2=lnM+12lnn0n02d02−1

Thus ${\mathrm{lnM}}^{\left[d_{0}\right]}=0$ exactly when $d_{\mathrm{eq}}=d_{0}$.

When $n_{0}$ is large, ${\mathrm{lnM}}^{*}\approx \ln\left(d_{0}/\sqrt{2}\right)$, so

$${\mathrm{lnM}}^{\left[d_{0}\right]}\approx \mathrm{lnM}+\ln\left(\frac{\sqrt{2}}{d_{0}}\right).$$

In particular, for $d_{0}=1$, ${\mathrm{lnM}}^{\left[1\right]}\approx \mathrm{lnM}+\ln\left(\sqrt{2}\right)$.

Importantly, (sampling) variance is invariant under this type of centering. Let $c_{i}=\frac{1}{2}\ln\left(\frac{n_{0i}}{\frac{n_{0i}}{2}\;d_{0}^{2}-1}\right)$. Since $c_{i}$ is a deterministic function of sample sizes, ${\mathrm{lnM}}_{i}^{\left[d_{0}\right]}={\mathrm{lnM}}_{i}+c_{i}$ has $\mathrm{Var}\left({\mathrm{lnM}}_{i}^{\left[d_{0}\right]}\right)=\mathrm{Var}\left({\mathrm{lnM}}_{i}\right)$ and identical inverse‐variance weights. Confidence intervals translate by $c_{i}$. In meta‐analysis, therefore, we could fit models on lnM and apply the offset $c_{i}$ post hoc to fitted means and intervals.

## Appendix D Why Not Use ln∣d∣ as a Magnitude Effect Size?

Given that the standardised mean difference (SMD) is widely used in meta‐analysis, it is natural to ask whether a magnitude analysis could be performed by working on a log scale with the absolute SMD, that is, ln∣d∣. At first glance, ln∣d∣ may appear attractive because it is unbounded above and thus might seem to provide a “Gaussian‐friendly” analysis scale. Here we show that, although ln∣d∣ has a simple delta‐method sampling variance, it is undefined at d=0. This boundary issue is a key reason we prefer lnM as our primary magnitude effect size in the main text.

Let d denote the study‐level standardised mean difference (Cohen's d), with estimated sampling variance $v_{d}=\mathrm{Var}\left(d\right)$ obtained from standard SMD theory for the relevant design. We define the log‐absolute SMD as:

(A37)
y≡ln∣d∣

This transform is undefined when d=0 and tends to −∞ as ∣d∣→0.

We treat y=ln∣d∣ as a smooth function of d for d≠0. For d≠0,

(A38)
$$\frac{\mathrm{dy}}{\mathrm{dd}}=\frac{d}{\mathrm{dd}}\ln|\;d\;|=\frac{1}{d}$$

A first‐order Taylor expansion (delta method) therefore gives:

(A39)
$$\begin{array}{c} \mathrm{Var}\left(\ln|d|\right)\approx {\left(\frac{1}{d}\right)}^{2}\mathrm{Var}\left(d\right)=\frac{v_{d}}{d^{2}},\;\;\mathrm{SE}\left(\ln|d|\right)\approx \frac{\sqrt{v_{d}}}{|\;d\;|} \end{array}$$

If one uses an alternative expression for $v_{d}$ (exact or approximate; independent‐groups or paired designs), the delta‐method mapping remains Equation (A39).

For two independent groups of sizes $n_{1},n_{2}$, a commonly used large‐sample approximation for the sampling variance of the SMD is.

(A40)
$$v_{d}\approx \frac{n_{1}+n_{2}}{n_{1}n_{2}}+\frac{d^{2}}{2\left(n_{1}+n_{2}-2\right)}$$

Substituting into Equation (A39) yields:

(A41)
$$\mathrm{Var}\left(\ln|d|\right)\approx \frac{n_{1}+n_{2}}{n_{1}n_{2}\;d^{2}}+\frac{1}{2\left(n_{1}+n_{2}-2\right)}$$

Other small‐sample or design‐specific formulas for $v_{d}$ can be used in the same way.

Equations ((A39), (A40), (A41)) highlight that ln∣d∣ behaves awkwardly when the standardised mean difference is close to zero. The first issue is definitional: ln∣d∣ does not exist at d=0 and it tends to −∞ as ∣d∣→0. In practice, this means that an analysis based on ln∣d∣ must adopt an explicit rule for handling d=0 (and very small ∣d∣), for example by excluding such cases or by applying a constant such as $\ln\sqrt{d^{2}+c}$ for some c>0.

The second issue concerns precision. From Equation (A39), the delta‐method variance is proportional to $1/d^{2}$, so the sampling variance on the ln∣d∣ scale increases rapidly as ∣d∣ approaches zero. Given that near‐null effects are common in empirical syntheses, the chosen handling rule near d=0 can affect the behaviour and interpretation of meta‐analytic models.

The motivation for lnM in the main text is to provide a log‐scale magnitude effect size without requiring an additional convention specifically to handle d=0. Like ln∣d∣, lnM has non‐trivial behaviour near its own boundary region (notably when $MS_{B}\leq MS_{W}$), but our framework makes that boundary explicit and provides a principled operational solution via SAFE. In this sense, Appendix D shows that ln∣d∣ offers a simple delta‐method sampling variance but is undefined at d=0, whereas lnM is designed to target a magnitude parameter while retaining a workable estimation strategy across the problematic boundary region.
